# A Path-Based Feature Selection Algorithm for Enterprise Credit Risk Evaluation

**DOI:** 10.1155/2022/7650207

**Published:** 2022-05-09

**Authors:** Marui Du, Yue Ma, Zuoquan Zhang

**Affiliations:** ^1^School of Science, Beijing Jiaotong University, Beijing 100044, China; ^2^Guanghua School of Management, Peking University, Beijing, China

## Abstract

In recent years, there has been increasing interest in exploring diversified features to measure small and medium-sized enterprises (SMEs) credit risk. Path-based features, revealing logical connections between SMEs, are widely adopted as informative feature kinds for causal inference in credit risk evaluation. Since there may exist thousands of feature paths to the target enterprise, to evaluate its credit risk, how to select the most informative path-based features becomes a challenging problem. To solve the problem, in this paper, we propose a novel method of feature selection, considering both similarity and importance on features' structured semantics as the factors of informativeness. With this, the proposed method can effectively rank both conventional and path-based features together. Furthermore, to improve the efficiency of the method, a heuristic algorithm is proposed to fast search for the candidate features. Through extensive experiments, we show our method performs competitively with other state-of-the-art selection methods.

## 1. Introduction

Small and medium-sized enterprise (SME) is an essential part of the national economy, whose development directly affects the growth of the country economy. In recent years, how to accurately assess the credit risk of SMEs attracts great attention from academy and industry. The most adopted approach is to evaluate the risk by incorporating various financial SME features to predict whether potential risks exist, based on some statistical methods. Among various kinds of features, conventional feature and path-based feature are two feature types commonly used in the process of evaluation.

**Figure 1:**
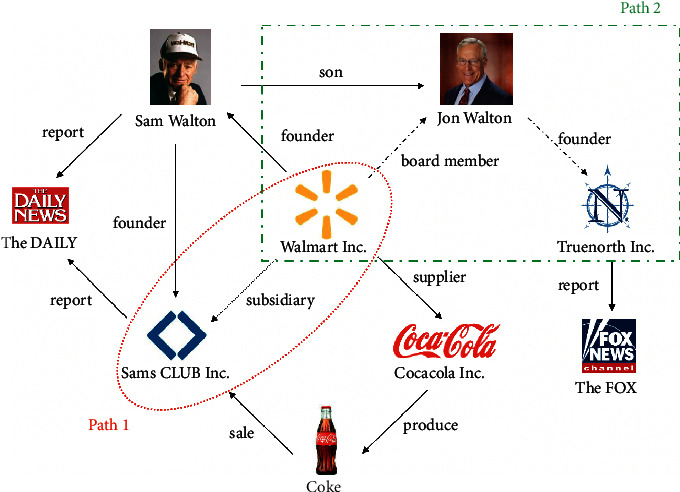
Example of Walmart information network.

Conventional features refer to unstructured and independent financial features, which reflect the basic information of enterprises. For example, the common conventional features include enterprise solvency, employee size, and business duration. Path-based features indicate well-structured and interdependent financial features, which describe the external influences to enterprises through specified relationships. For example, in Figure 1, path 1 is a path feature representing there is a parent-subsidiary relation between Walmart and Sams CLUB.

Conventional features mainly focus on describing enterprises' self-related information, which may be a bit ineffective to evaluate the credit risk in today's financial environment. The reason is that, with the expansion of the global market size, SMEs usually have a large amount of complicated relations with other SMEs, and their financial status can be easily affected by their related SMEs, which makes simple self-related features lose their effectiveness. For example, an SME may still have potentially high risk even it is in good financial conditions since the contagion risk may come from its associated enterprises, such as its parent enterprises. Therefore, compared to self-related information, interaction information between SMEs should be paid more attention in studying SME credit risk. Path-based feature is proposed to model such interactions in the information networks [1]. To not lose important information, heterogeneous information networks [2] are often used to model SME complicated relations with graph data structure. In the network, every specified relation between two enterprises can be represented as one graph path, whose semantic information can be explicitly captured from the data structure. For example, in Figure 1, Path 2 represents the information that Truenorth's founder is also the board member of Walmart. If Truenorth is in financial crisis, then it may affect the financial status of Walmart. In this way, complicated relations between SMEs can be systematically and concisely defined in graph paths.

Even though path-based features demonstrate the advantage on evaluating credit risk, in SME information networks, there may exist numerous paths to an enterprise, some of which may carry useless information for evaluation. Thus, how to select the most informative features becomes a challenging problem. Unfortunately, most existing methods of feature selection may not apply well for path-based features since they are originally designed for conventional features which never consider the structure semantics of features. If these methods are used for path-based features, many features with similar structured semantics will be retained which makes the candidate feature set focus too much on limited information. Therefore, in this paper, we propose a novel feature selection method, considering both importance and similarity on features' structured semantics as the factors of informativeness. First, we measure a feature's importance based on its classification performance using some supervised classifier. The features contributing greatly to classify default SMEs are regarded as important features. Next, besides the importance, the similarity between candidate features is taken as another essential factor to consider in our selection method. To keep selected features unique and diversified, we introduce two kinds of measures to evaluate similarity between features, for the purpose of reducing feature redundancy. One measure focuses on the similarity of classification result, and the other focuses on the similarity of path structure. At last, to improve the efficiency of the proposed method, a heuristic selection algorithm is used to accelerate the selection process. Both theory and practice show the algorithm can greatly speed up the selection process and achieve satisfied selection results.

In the rest of this paper, Section 2 introduces the SME credit risk evaluation methods and the state-of-the-art feature selection methods; Section 3, gives the basic information of information network and the commonly used path-based features. In section 4, we propose a novel feature selection method and introduce a heuristic algorithm to accelerate the selection process.  Section 5 presents the experiment and analysis of the experimental results, and Section 6 concludes the paper.

## 2. Related Work

In the 1960s, Altman [3] used a set of financial features to evaluate enterprise credit risk. Since then, many researchers have focused on using financial features to evaluate SME credit risk. For example, Cultrera [4] used the current ratio, total asset turnover rate, and ten more financial ratios to evaluate SME credit risk. Gupta [5] investigated the effectiveness of operating cash flow for UK SMEs. The financial features can provide meaningful SME situations. However, due to the imperfect internal system of enterprises, the financial statements of many SMEs may be unaudited and unreliable. Thus, many researchers start to add nonfinancial features to the evaluation system such as enterprise age [6], industrial sector [7], the ability of enterprise managers [8], and enterprise management structure. Tsai [9] used enterprise news information on the credit risk of SMEs. Yin [10] used SME legal judgment information with financial and firm nonfinancial features to evaluate credit risk. With the development of data mining strategy, data related to enterprises have been accumulated such as the upstream and downstream enterprise information and the parents or subsidiary enterprise information. Numerous relationships between different entities have also provided researchers with new ideas to find SME credit risk factors. Several researchers use information networks to extract SME-related features. For example, Moro [11] takes the impact of SMEs and bank manager trust relationship on enterprise credit risk into consideration. Tobback [12] collects interenterprise relationship data to measure SME credit risk. Kou [13] collects enterprise manager, shareholder, and payment information and builds three information networks to extract evaluation features. However, due to the complicated relationships between SMEs and their associated entities, some essential information may be lost by only considering homogeneous relations. Therefore, many researchers extent the object and relation types between SMEs and their associated entities. Du [14] collects enterprise, person, commodity, and news information of SMEs and builds an information network of SMEs to measure credit risk. Zhong [15] collects enterprise, investor, enterprise category, and enterprise location and builds an information network to make investment behavior prediction. Extracting enterprise-related information through information networks dramatically increases the number of features used to measure enterprise credit risk.

Feature subset generation methods can be divided into three categories. The first one refers to complete search strategy [16], which determines feature subset by finding all combination possibilities. The second one refers to the heuristic search strategy [17], which evaluates each search location to get the best one and then searches from this location until reaching the goal. This method avoids a large number of unnecessary search paths, reduces the amount of calculation, and improves efficiency. The third one refers to the random search strategy [18], which randomly generates a number of feature subsets and then evaluates these feature subsets. Feature subset evaluation method mainly includes two types: class relevance and remove redundancy. Most feature subset evaluation methods can find the most relevant features effectively. For example, the Relief [19] and ReliefF [20] algorithms. However, it is unable to remove redundant features. Therefore, many feature selection algorithms are proposed, such as the mRMR algorithm [21], and information theory is applied to measure both class relevance and pairwise correlation between features. The FCBF [22] applies symmetrical uncertainty to measure both class relevance and pairwise correlation between features. Furthermore, the relationship between features is complex. Some feature subset evaluations consider class relevance, feature redundancy, and complementarity. The RCDFS [23] extends the traditional redundancy analysis to redundancy-complementariness analysis other than the class relevance and redundancy measures. The self-adaptive feature evaluation (SAFE) [24] algorithm applies the complement strategy in the process of searching and proposes an adaptive cost function to penalize redundancy and reward complementary. This paper proposes a feature selection algorithm that considers class relevance, feature redundancy, and feature structures and semantics.

## 3. Preliminary

Information network is a classical data structure used to model objects and relations in a directed graph. Given different objects in information networks, logical connections can be effectively constructed, and semantic relationships can be easily captured.

**Definition 1 .**

An information network defined as a directed graph *G*=(*𝒱*, *ℰ*) with object type function *τ* : *𝒱*⟶*𝒜* and relation type function *ϕ* : *ℰ*⟶ℛ, where object *v* ∈ *𝒱* belongs to object type *τ*(*v*) ∈ *𝒜* and link *e* ∈ *ℰ* belongs to relation type *ϕ*(*e*) ∈ ℛ.

**Figure 2:**
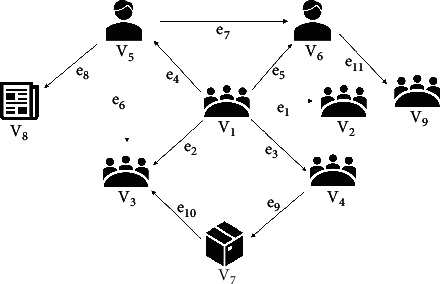
Example of information network.

Figure 2 is an example of information network for enterprise *v*_1_.

In this network, it contains four object types *𝒜*: enterprise (*𝒜*_*e*_), commodity (*𝒜*_*c*_), person (*𝒜*_*p*_), and news (*𝒜*_*n*_). And, eight relation types ℛ: ℛ_subsidiary_, ℛ_supplier_, ℛ_report_, ℛ_founder_, ℛ_produce_, ℛ_board member_, ℛ_son_, and ℛ_sale_. From the graph, objects *v*_1_, *v*_2_, *v*_3_, *v*_4_, and *v*_9_ are enterprise, that we have *τ*(*v*_1_)=*𝒜*_*e*_, the same as *τ*(*v*_2_), *τ*(*v*_3_), *τ*(*v*_4_),  and *τ*(*v*_9_) are. Objects *v*_7_ is commodities, that we have *τ*(*v*_7_)=*𝒜*_*c*_. Objects *v*_8_ is news, that we have *τ*(*v*_8_)=*𝒜*_*n*_. Objects *v*_5_ and *v*_6_ are persons, that we have *τ*(*v*_5_)=*𝒜*_*p*_, the same as *τ*(*v*_6_). *e*_1_ and *e*_2_ are the relation of subsidiary, that we have *ϕ*(*e*_1_)=ℛ_subsidiary_, the same as *ϕ*(*e*_2_). *e*_3_ is the relation of supplier, that we have *ϕ*(*e*_3_)=ℛ_supply_. *e*_4_, *e*_6_, and *e*_11_ are the relation of founder, that we have *ϕ*(*e*_4_)=ℛ_founder_, the same as *ϕ*(*e*_6_), *ϕ*(*e*_11_) are. *e*_5_ is the relation of board member, that we have *ϕ*(*e*_5_)=ℛ_board member_. *e*_7_ is the relation of son, that we have *ϕ*(*e*_7_)=ℛ_son_. *e*_8_ is the relation of reports, that we have *ϕ*(*e*_8_)=ℛ_report_. *e*_9_ is the relation of produce, that we have *ϕ*(*e*_9_)=ℛ_produce_. *e*_10_ is the relation of sale, that we have *ϕ*(*e*_10_)=ℛ_sale_.

**Definition 2 .**

The network schema *S*=(*𝒜*, ℛ) is a metalevel representation for *G*=(*𝒱*, *ℰ*) with object type function *τ* : *𝒱*⟶*𝒜* and relation type function *ϕ* : *ℰ*⟶ℛ, which is a directed graph over object types *𝒜* and edges as relations from ℛ.

**Figure 3:**
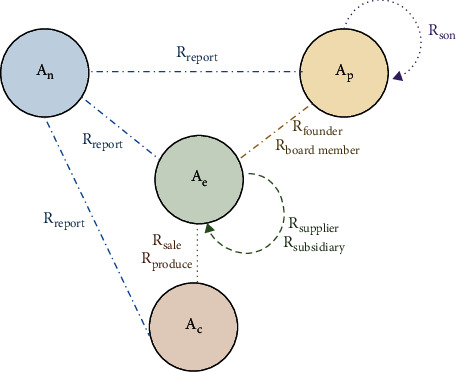
The network schema.

Figure 3 shows the corresponding network schema of Figure 2.

**Definition 3 .**

With a schema *S*=(*𝒜*, ℛ), a path *P* in the form ${𝒜}_{1}\overset{{ℛ}_{1}}{⟶}{𝒜}_{2}\overset{{ℛ}_{2}}{⟶}\ldots \overset{{ℛ}_{n}}{⟶}{𝒜}_{n+1}$ which defines a composite relation ℛ=ℛ_1_°ℛ_2_°…°ℛ_*n*_ between *𝒜*_1_ and *𝒜*_*n*+1_, where ° denotes the composition operator on relations. For simplicity, we use the names of object types and relation types denoting the path: *P*=*𝒜*_1_*∗*ℛ_1_*∗𝒜*_2_ … ℛ_*n*_*∗𝒜*_*n*+1_.

From the above definitions, some commonly used path-based features are given:

1. Common-neighbors Feature [25]: common-neighbors feature is defined as the number of common neighbors shared by two objects *x*_*i*_ and *x*_*j*_, namely, |Γ(*x*_*i*_)∩Γ(*x*_*j*_)|, where Γ(*x*) is the notation for neighbor set of the object *x* and |·| denotes the size of a set.
2. Path-count feature [26]: path-count feature is defined as the number of path instances between two objects *x*_*i*_ and *x*_*j*_ following a given metapath *P*, denoted as *PC*_*P*_(*x*_*i*_, *x*_*j*_).
3. Naive-MP feature [14]: Naive-MP feature is defined as the impact of meta path *P* on target object, denoted as *N*_*P*_(*x*)=|{*x*′ ∈ *D|*∃ *p*_*x*⇝*x*′_ ∈ *P*, Γ(*x*′)=1}|/|{*x*′ ∈ *D|*∃ *p*_*x*⇝*x*′_ ∈ *P*}|, where *D* is an SME object collection, *p*_*x*_*i*_⇝*x*_*j*__ is a path instance from object *x*_*i*_ to object *x*_*j*_, and Γ(*x*) is the risk inference function defined in [14].

In Figure 2, we can see that *v*_1_ has 2 paths in the form *𝒜*_*e*_*∗*ℛ_subsidiary_*∗𝒜*_*e*_, which are $v_{1}\overset{e_{1}}{⟶}v_{2}$ and $v_{1}\overset{e_{2}}{⟶}v_{3}$. To illustrate path-based features, we take path-count feature as example. When evaluating the credit risk of *v*_1_, we can have its path-count feature on the path *𝒜*_*e*_*∗*ℛ_subsidiary_*∗𝒜*_*e*_ equals to 2, which means that the enterprise *v*_1_ totally has 2 subsidiaries.

## 4. Methods

In this section, a method is proposed to find the top-k informative features from the pool of candidate features. Regarding candidate features have high importance on predicting default SME and low similarity on classification result and path structure, as the informative ones. The measurement of importance and similarity will be detailed, respectively, in Section 4.1 and Section 4.2. The final set of top-k features will be selected in Section 4.3.

### 4.1. The Importance of Features

An important feature is a feature that has a significant impact on determining whether an enterprise is default. It helps direct our model to learn and predict correctly. In this paper, we measure a feature's importance based on its classification performance using some supervised model. Based on the classification result from the supervised model, we can evaluate the given feature in different measures such as accuracy, precision, recall, and *F*_1_. Specifically for the SME default problem, the datasets are usually highly imbalanced, where the number of default enterprises is much less than the number of nondefault enterprises. In order to correctly find default enterprises as many as possible, we select *F*_1_ as the importance measure which can balance the effect of both precision and recall. For simplicity, the logistic regression model [27] is used as the supervised model in this paper. The definition of *F*_1_ measure is given as follows.

**Definition 4 .**

(1) $$\begin{array}{c} F_{1}=\frac{2*\text{precision}*\text{recall}}{\text{precision}+\text{recall}},\\ \text{recall}=\frac{\left|\left\{\left(x,y\right)\in D|y=1,h\left(x\right)=1\right\}\right|}{\left|\left\{\left(x,y\right)\in D|y=1,h\left(x\right)=1\right\}\right|+\left|\left\{\left(x,y\right)\in D|y=1,h\left(x\right)=0\right\}\right|},\\ \text{precision}=\frac{\left|\left\{\left(x,y\right)\in D|y=1,h\left(x\right)=1\right\}\right|}{\left|\left\{\left(x,y\right)\in D|y=1,h\left(x\right)=1\right\}\right|+\left|\left\{\left(x,y\right)\in D|y=0,h\left(x\right)=1\right\}\right|}, \end{array}$$

where *x* is an enterprise in the dataset *D*, *y* is the actual status of *x*, *h*(*x*) is the predicted status of *x*, *y*=1 means *x* is default, and *y*=0 means *x* is nondefault.

The value of *F*_1_ measure is used as the score of the feature importance. In the rest of this paper, we denote the importance score of feature *f* as imp(*f*).

### 4.2. The Similarity between Features

Besides the importance of features, the similarity between features is another essential factor to consider in the process of feature selection. Similar features may bring redundancy to the selection result, making the selected features focus too much on limited information. With the redundant features, the learned model may lose its generalization ability on classification. In order to keep the model effective, we expect the selected features as mutually different as possible. In the next, we introduce two measures to evaluate the similarity between features. The first one is based on the consistency of classification results. The second one is based on the matching of path structure.

#### 4.2.1. Similarity on Classification Result

The importance measure evaluates each feature based on its individual classification performance. However, it is possible that two features have the same importance score but different predictions on some data examples. The difference measures how far two features can come to an agreement on the status of an enterprise. The less the difference, the less the similarity of the views shared by those features. Thus, the consistency of features' classification results can be treated as a similarity measure. In this paper, the consistency between features is computed through the classification result learned from the supervised model, which is similar to the process of computing feature importance. That is, we use each feature to train a logistic regression model to classify default SMEs, and the consistency of results is taken as the similarity between features. We formally define the mentioned consistency similarity as follows.

**Definition 5 .**

(2) $$\begin{array}{c} {\text{Sim}}_{\text{cls}}\left.\left(f,f^{\prime }\right)\right)=\frac{\left|\left\{x\in D|h_{f}\left(x\right)=h_{f^{\prime }}\left(x\right)\right\}\right|}{\left|\left\{x\in D\right\}\right|}, \end{array}$$

where *x* is an enterprise in the dataset *D* and *h*_*f*_(*x*) and *h*_*f*′_(*x*) are the predicted status of *x* by the supervised model learnt respectively from feature *f* and feature *f*′.

According to the definition, Sim_cls_(*f*, *f*′) is exactly the similarity between the features on their classification results.

#### 4.2.2. Similarity on Path Structure

In the above, the consistency of classification is used to measure the similarity between features. However, this measure is a bit biased as its result may vary with different business backgrounds. For instance, when studying SMEs of conventional retail, we may see that the similarity between the feature of product quality and the feature of marketing director capability is relatively high, and both of them are essential factors in default prediction; conversely, when studying SMEs of online retail, we may see that the similarity between those two features may decrease since e-commerce enterprises usually are significantly product-driven rather than marketing-driven. In order to alleviate such bias, we hereby introduce another measure to evaluate feature similarity from the perspective of semantics, which is naturally independent of business backgrounds. We regard the similarity of path structure as the exact similarity of the features semantics. The high diversity of paths improves the compatibility and the robustness of the learned model. Mathematically, we use Levenshtein distance [28] to measure the similarity between paths. The distance is the least step in changing a path to another path. We denote the mentioned similarity as *Sim*_*path*_, and the definition is given as follows:

**Definition 6 .**

(3) $$\begin{array}{c} {\text{Sim}}_{\text{path}}\left(f,f^{\prime }\right)=\frac{\max\left(\text{len}\left(P_{f}\right),\text{len}\left(P_{f^{\prime }}\right)\right)-\text{lev}\left(P_{f},P_{f^{\prime }}\right)}{\max\left(\text{len}\left(P_{f}\right)\text{,len}\left(P_{f^{\prime }}\right)\right)}, \end{array}$$

where *P*_*f*_ and *P*_*f*′_ are the path structures of feature *f* and feature *f*′, len(*P*_*f*_) and len(*P*_*f*′_) are the path lengths of *P*_*f*_ and *P*_*f*′_, and lev(*P*_*f*_, *P*_*f*′_) is Levenshtein distance between the two features.

For example, according to our method, the path structure to the feature of one enterprise's marketing director capability is *𝒜*_*e*_*∗*ℛ_control_*∗𝒜*_*p*_ and to the feature of one enterprise's product quality is *𝒜*_*e*_*∗*ℛ_produce_*∗𝒜*_*c*_. Computing the distance between the two path structures is actually to compute Levenshtein distance between the two path structures. With the result distance 2, we can have the similarity on path structure between the two features is 0.33.

### 4.3. The Proposed Feature Selection Algorithm

With the measures of importance and similarity, in this section, we give an algorithm to find the top-k informative features. Each feature we select should have a high importance score and low similarity scores with other features. That is to say, the final feature set we select should have maximum total importance score and minimum total similarity score among all the possible feature combinations from the candidate feature pool. The mathematical goal can be presented as follows:

(4) $$\begin{array}{c} \underset{C}{\max}\sum_{f\in C}\text{imp}\left(f\right)-\sum_{f,f^{\prime }\in C}^{f\neq f^{\prime }}\left(\alpha \cdot {\text{Sim}}_{\text{cls}}\left(f,f^{\prime }\right)+\beta \cdot {\text{Sim}}_{\text{path}}\left(f,f^{\prime }\right)\right),\\ s.t\;C\subseteq S,\left|C\right|=k,\left|S\right|=m. \end{array}$$

where *S* is the pool of all candidates features with size *m*, *C* is the result set of selected features with size *k*, and *α* and *β* are two weight parameters of Sim_cls_(*f*, *f*′) and Sim_path_(*f*, *f*′) with features *f* and *f*′.

It is obvious that exhaustive searching is inappropriate to solve above problem, whose time complexity is *O*(*C*_*m*_^*k*^). When the number of features is large, the process of searching is significantly time-consuming. Usually, greedy searching algorithms are applied on this problem. However, for naive greedy algorithm, as long as one feature is not selected into the result set, its similarity with other features already selected will be calculated repeatedly at each iteration. Such computation on similarity is wasteful. Therefore, we propose an upgraded version, a greedy-search feature selection (GSFS) algorithm (in Algorithm 1), to find the result set. Our proposed algorithm is a practical greedy algorithm with the time complexity of *O*(*mk*).

The proposed algorithm always can find the local optimal solution in the process of feature selection. The proof and analysis are given in the rest of this section.

**Theorem 1 .**

Through the searching algorithm 1, the local optimal solution to (4) can be always found.

**Proof**

As a greedy searching algorithm always looks for local optimal solution based on its previous result, it indicates that when a new feature is selected, and the previous selected features are kept. Then, at the (*t*+1)-th iteration, there must exist *C*_*t*_ ⊂ *C*_*t*+1_, and the objective of greedy can be rewritten as

(5) $$\begin{array}{c} \underset{f\in S_{t}}{\max}\left(\sum_{g\in C_{t}}\text{imp}\left(g\right)-\sum_{g,g^{\prime }\in C_{t}}\left(\alpha \cdot {\text{Sim}}_{\text{cls}}\left(g,g^{\prime }\right)+\beta \cdot {\text{Sim}}_{\text{path}}\left(g,g^{\prime }\right)\right)+\text{imp}\left(f\right)-\sum_{f^{\prime }\in C_{t}}\left(\alpha \cdot {\text{Sim}}_{\text{cls}}\left(f,f^{\prime }\right)+\beta \cdot {\text{Sim}}_{\text{path}}\left(f,f^{\prime }\right)\right)\right). \end{array}$$

As the first part of the objective is the result achieved at the *t*-th iteration, it becomes constant at the *t*+1-th iteration. Therefore, maximizing the objective in (5) is to maximize its second part:

(6) $$\begin{array}{c} \underset{f\in S_{t}}{\max}\left(\text{imp}\left(f\right)\right.-\sum_{f^{\prime }\in C_{t}}\left(\alpha \cdot {\text{Sim}}_{\text{cls}}\left(f,f^{\prime }\right)+\beta \cdot {\text{Sim}}_{\text{path}}\left(f,f^{\prime }\right)\right). \end{array}$$

With notations in the Algorithm 1, maximizing the second part is equal to maximize the following:

(7) $$\begin{array}{c} \underset{f\in S_{t}}{\max}\theta_{f}-\sum_{f^{\prime }\in C_{t}}\eta_{f,f^{\prime }}. \end{array}$$

In Algorithm 1, with the selected feature *f*^*∗*^ at each iteration, the algorithm iteratively updates *w*_*f*_ of each *f* in the current candidate feature set with *w*_*f*_=*w*_*f*_ − *η*_*f*,*f*^*∗*^_. It can be obviously seen that, for *f* not yet selected, *w*_*f*_=*θ*_*f*_ at the 1-st iteration. At the 2-nd iteration, *w*_*f*_=*θ*_*f*_ − ∑_*f*′∈*C*_1__*η*_*f*,*f*′_ and at the (*t*+1)-th iteration *w*_*f*_=*θ*_*f*_ − ∑_*f*′∈*C*_*t*__*η*_*f*,*f*′_. Therefore, in Algorithm 1, we can have

(8) $$\begin{array}{c} f^{*}=\text{arg}\underset{f\in S}{\max}w_{f},\\ =\text{arg}\underset{f\in S}{\max}\theta_{f}-\sum_{f^{\prime }\in C_{t}}\eta_{f,f^{\prime }}. \end{array}$$

Selecting *f*^*∗*^, the feature of the maximum *w*_*f*_ at each iteration, is equivalent to selecting the feature that satisfies the objective in (6). The theorem proves.

## 5. Experiments

In this section, we are going to investigate the effectiveness of our proposed method. We conduct experiments on three real-world datasets. The result and explanation will be detailed in this section.

### 5.1. Experimental Settings

In our experiments, three datasets are used for comparison. SMB1 dataset provides the information of traditional small and medium-sized enterprises. GEM2 and STAR3 datasets give the statistics about high technology enterprises. All the datasets can be downloaded from CSMAR4. 48 frequently used conventional features, and 4548 path-based features are used for feature selection. The statistics of datasets is shown in Table 1.

All the experiments were implemented in Python 2.7.17 on Win 8.1+ with CPU *i*5 − 9300+ processor and 8*G*+ RAM.

### 5.2. Performance of Feature Selection

In this section, we compare our proposed method with five state-of-the-art selection methods for ranking the most informative features. For our method, for different datasets, *α* and *β* are configured according to the settings in Section 5.3, respectively. The details of the other five selection methods are introduced as follows:

  mRMR [21]: a very famous feature selection algorithm that applies mutual information (MI) metrics to measure feature-class relevance and pairwise correlation between features
  FCBF [22]: it first applies symmetrical uncertainty (SU) as a metric to measure feature-class relevance and then uses an approximate Markov blanket to check redundant features
  mIMR [29]: it considers feature-class relevance and the net effect of redundancy and complementarity, using joint mutual information
  RCDFS [23]: it not only considers feature-class relevance and pairwise correlation between features, but also takes into account the effect of redundancy-complementariness dispersion
  FS-RRC [30]: first applies symmetrical uncertainty (SU) as a metric to measure feature-class relevance and then uses an approximate Markov blanket to check redundant features, and finally the complementary score between features based on both SU score and MI

**Figure 4:**
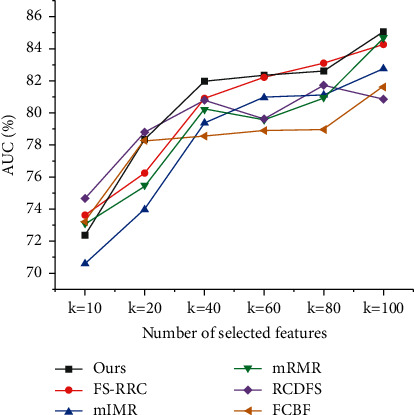
The ROC curves for SMB dataset.

**Figure 5:**
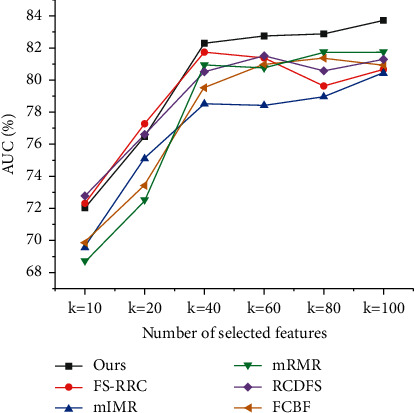
The ROC curves for GEM dataset.

**Figure 6:**
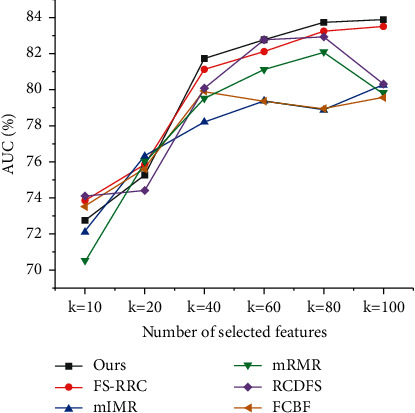
The ROC curves for STAR dataset.

All comparisons are conducted on the mentioned three datasets. To compare mentioned methods, 10-fold cross-validation associated with the logistic regression is used to evaluate their performance. Specifically, we divide the datasets into ten folds, using nine folds for training and one for testing. Then we repeat the cross-validation 20 times, calculating the classification accuracy and AUC of each mentioned method. In order to compare feature selection methods comprehensively, we, respectively, do experiments with *k*=20, *k*=40, and *k*=80, where *k* represents the number of features to select. The comparison results are summarized in Figures 4–6 and Tables 2 and 3.

From the above results, we can see that, in most cases, our proposed feature selection method has better performance than other five selection methods. Although the other five methods also remove similar features using different similarity measures, none of them consider the similarity of feature semantics, making their results not as concise as ours. For example, in the dataset GEM, *𝒜*_*e*_*∗*ℛ_supplier_*∗𝒜*_*e*_ path feature and *𝒜*_*e*_*∗*ℛ_supplier_*∗𝒜*_*e*_*∗*ℛ_sale_*∗𝒜*_*c*_ path feature are both selected by all other five methods; however, our method only picks *𝒜*_*e*_*∗*ℛ_supplier_*∗𝒜*_*e*_*∗*ℛ_sale_*∗𝒜*_*c*_ path feature and ignores *𝒜*_*e*_*∗*ℛ_supplier_*∗𝒜*_*e*_ path feature since *𝒜*_*e*_*∗*ℛ_supplier_*∗𝒜*_*e*_*∗*ℛ_sale_*∗𝒜*_*c*_ path feature has a high semantic similarity with *𝒜*_*e*_*∗*ℛ_supplier_*∗𝒜*_*e*_ path feature. With capturing the similarity of feature semantics, the feature redundancy of our result is lower than that of other result. 30% features selected by those methods are highly similar with path-based similarity scores larger than 0.7, but only 8% features of ours have that large similarity scores.

In Table 2, for SMB dataset, it is interesting to see that most methods have similar AUC scores in the setting *k*=20, but when *k*=40 or *k*=80, our method outperforms the other five methods. The reason is that, for some complex dataset like SMB, when only 20 features can be selected, all methods perform similarly poor without enough features for classification, but when 40 or 80 features can be selected, the methods have enough quota to demonstrate different mechanics to pick features and achieve different performance. The main difference between the results of compared methods comes from their different similarity measures to filter redundant features. In the setting *k*=80, we can see that the other five methods finally have 55 features in common, but our method only have 20 same features with them. As the compared methods are not originally designed for path-based features, it is not strange that they select many similar path-based features. But for our method, by considering the semantic similarity of path-based features, we can efficiently eliminate the redundancy of selected features, making our method hold an 2.52% AUC lead over other methods in SMB dataset.

### 5.3. Combination of Parameters

In this section, for our method, we will run experiments to compare the effects of different parameter combinations. Our proposed method mainly has two key parameters, *α* and *β*, which need to be carefully determined. *α* controls the weight of the classification similarity, and *β* controls the weight of the path-structure similarity.  Table 4 shows the classification accuracy of our method with different parameter combinations in the three datasets.

From the table, it can be observed that, for SMB dataset, the setting *α*=0.3 and *β*=0.7 performs best; for GEM dataset, the setting *α*=0.4 and *β*=0.6 performs best; for STAR dataset, the setting *α*=0.8 and *β*=0.2 performs best. It is interesting that, for different datasets, the optimal parameter combinations differ greatly. The reason may be that the complexity of SME relations in the three datasets is in different level. To the dataset STAR, as there exist only 2157 possible path patterns and most of which are simple and short, the path-structure similarity does not play a big role in reducing redundancy. However, to dataset SMB and GEM, as more complicated path patterns are contained in the datasets, it becomes necessary to exploit the path-structure similarity to filtering redundant features. Therefore, in our experiments, different parameter combinations of *α* and *β* are set, respectively, for the different datasets.

### 5.4. Efficiency Analysis

**Figure 7:**
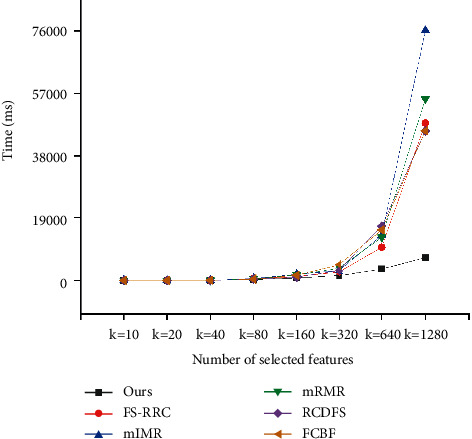
Computation time comparison for SMB dataset.

**Figure 8:**
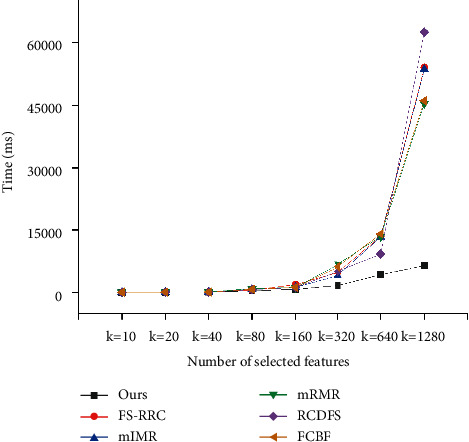
Computation time comparison for GEM dataset.

**Figure 9:**
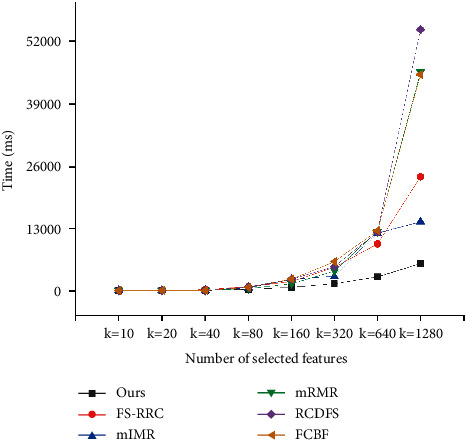
Computation time comparison for STAR dataset.

In this section, efficiency experiment is conducted to show our method can perform rapidly. To compare efficiency, we run all the methods on the three datasets and record the running time of finding *k* features. From Figures 7–9, it can be obviously seen that our method runs fastest among all the methods on the three datasets. Take experiments on the dataset GEM as illustration. When *k*=20, our method outperforms other methods with 20 ms at least; when *k*=160, our method outperforms others with 417 ms at least; and when *k*=640, our method outperforms others with 4928 ms at least. It is easy to see that, with *k* increasing larger, the difference of performance between our method and others becomes greater as well. The reason is that the other five methods run to select features in an exhaustive way, whose time complexity grows exponentially with the value of *k*; however, our method presented in Algorithm 1 runs to select features in a heuristic way, whose time complexity grows linearly with the value of *k*. Therefore, in practice, we can clearly find that the efficiency of our method far exceeds those of other methods in general. Overall, the results shown in Sections 5.2 and 5.3 demonstrate that compared to the other methods, our method has the capability to find features of higher quality with higher efficiency.

## 6. Conclusion

In this paper, we propose a novel method of feature selection, considering both importance and similarity. We first measure the importance of features based on their performance on identifying default SMEs. Then, the similarity of classification performance and the similarity of structure semantics are considered to reduce the redundancy of selected features. To improve the efficiency of our method, we also introduce a heuristic algorithm to accelerate the selection process. At last, empirical results demonstrate that our proposed method outperforms other state-of-the-art methods in feature quality and algorithm efficiency.

## Figures and Tables

**Algorithm 1:**
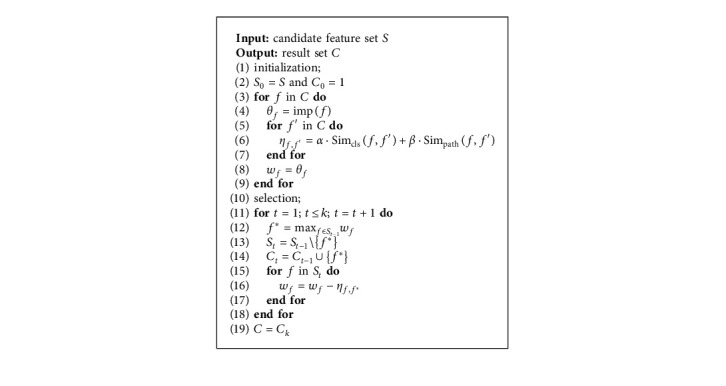
GSFS Algorithm.

**Table 1:** Dataset information.

	SMB	GEM	STAR
Number of enterprises	722	528	297
Number of people	96271	75628	57368
Number of news	38775	23098	9672
Number of commodities	26725	21893	1053
Number of path-count features	1324	1038	732
Number of common-neighbor features	967	1073	589
Number of Naive-MP features	1492	1384	836

**Table 2:** AUC score (%) comparison for three datasets

	Ours	FS-RRC	mIMR	mRMR	RCDFS	FCBF
SMB						
*k* = 20	75.26	75.85	**76.31**	76.05	74.42	75.59
*k* = 40	**81.73**	81.12	78.21	79.52	80.09	79.93
*k* = 80	**83.75**	83.23	78.86	82.10	82.93	78.95
GEM						
*k* = 20	76.47	**77.28**	75.10	72.53	76.61	73.42
*k* = 40	**82.30**	81.74	78.51	80.95	80.53	79.52
*k* = 80	**82.89**	79.63	78.95	81.76	80.58	81.37
STAR						
*k* = 20	78.37	76.25	73.97	75.48	**78.79**	78.29
*k* = 40	**81.99**	80.92	79.37	80.22	80.79	78.56
*k* = 80	**82.62**	82.10	81.11	80.96	81.73	78.97

**Table 3:** Classification accuracy (%) comparison for three datasets.

	Ours	FS-RRC	mIMR	mRMR	RCDFS	FCBF
SMB						
*k* = 20	90.50	90.52	90.47	89.47	90.35	89.92
*k* = 40	**89.47**	86.95	87.34	88.64	89.18	88.63
*k* = 80	**89.92**	89.54	87.39	88.78	89.20	89.11
GEM						
*k* = 20	90.81	89.05	90.46	88.81	**91.25**	90.06
*k* = 40	**88.35**	86.79	84.45	86.52	85.83	86.70
*k* = 80	**87.77**	87.71	86.72	85.81	86.45	84.74
STAR						
*k* = 20	87.56	87.47	**90.10**	85.50	87.03	87.52
*k* = 40	**85.88**	85.83	82.92	83.08	84.47	85.17
*k* = 80	**87.91**	87.35	86.06	84.86	87.71	84.73

**Table 4:** Classification accuracy (%) of different *α* and *β* combinations for three datasets.

*α*/*β*	0.1/0.9	0.2/0.8	0.3/0.7	0.4/0.6	0.5/0.5	0.6/0.4	0.7/0.3	0.8/0.2	0.9/0.1
SMB									
*k* = 20	84.96	85.33	**90.50**	88.56	89.92	85.96	73.97	75.10	70.53
*k* = 40	79.92	87.84	**89.47**	89.08	86.52	87.07	84.85	79.47	67.38
*k* = 80	85.44	88.21	**89.92**	89.63	89.64	86.82	80.73	76.52	68.09

GEM									
*k* = 20	75.87	82.11	86.06	**90.81**	87.35	90.07	89.92	79.96	79.46
*k* = 40	86.04	87.79	86.82	**88.35**	87.92	86.32	83.90	75.91	70.55
*k* = 80	83.81	84.91	83.59	**87.77**	87.48	84.72	86.57	79.73	79.92

STAR									
*k* = 20	77.26	67.29	78.64	78.18	80.78	83.69	85.85	**87.56**	86.77
*k* = 40	69.61	69.09	77.27	78.76	82.06	82.21	82.14	**85.88**	84.97
*k* = 80	69.72	65.36	75.36	71.55	82.03	81.81	85.75	**87.91**	87.23

## Data Availability

The data used to support the findings of this study are available from the corresponding author upon request.
